# Polar Cold—Probable Fate of Sir John Franklin

**Published:** 1852-02

**Authors:** 

**Affiliations:** Surgeon U. S. Navy


*Polar Cold. Probable Fate of Sir John Franklin.* By Dr. Kane, Surgeon U. S. Navy.

Dr. Kane, Surgeon to the late American Arctic Expedition, has just concluded a course of three lectures before the *Smithsonian Institution*, at Washington, relative to the voyage and researches of the gallant party who braved the perils of the Arctic regions in search of Sir John Franklin. We are sure we could not present to our readers anything more interesting than the following extracts from Dr. Kane's lectures:

We left New York, a united little body of thity-seven officers and men in the brigantines *Advance* and *Rescue*, on the 23d of May, 1850. Twenty-five days afterwards, we sighted the rugged mountains of Greenland, and by the 7th of July, found ourselves fast in the great ice-pack of Baffin's Bay.

The Bay of Baffin serves as the great thoroughfare of the Polar ice, on its passage to the South from the far Northern estauries which lead to the Arctic ocean. During the long winter the whole of this great Bay may be looked upon as one field of ice, which, whether moving or consolidated, is known technically as "the pack." This great body of ice does not end here. After throwing out innumerable processes into the *Fiords* of Greenland and America, it unites with a similar mass in Hudson's Bay, passes down the coast of Labrador, and even abuts against the Northern coasts of Newfoundland and the Straits of Belle Isle. This immense area—equal to the United States East of the Alleghanies—has annual variations in extent and condition. Influenced by winds and temperature, sometimes it is one enormous agglutination, sometimes a drifting chaos, composed of grinding fragments, varyidg in diameter from mere "skreed" i. e. rubbish, to "fields" many miles in diameter. Among these, with terrific crash and turmoil, the "ice mountains" pursue their resistless march.

The thermometer was here at the midsummer temperature of 2° above the freezing point; indeed *ice* formed freely during those hours of "low sun," known as night in that latitude. Yet the skies were warm and sunny, and the weather to our acclimated perceptions, worthier of the Bay of Naples than of Baffin. Here, too, the bergs were numerous, and the phenomena of reflection upon a scale of marvellous splendor.

Griffith's Island was the greatest westing, the greater barrier of ice beyond preventing further progress. The ice was gathering rapidly around them. The thermometer fell to but 3° above zero, and ice formed rapidly whenever the sea was at rest. By the morning of the 14th of September, the squadron was frozen up, fast in winter ice. The habitual rule of Arctic explorers is to seek a winter harbor; the present was the first recorded example of vessels caught in the open sea. Soon after the great sea of ice was in motion, northward, carrying, of course, its prisoners with it. Soon the commotion of the ice prevented fires. The thermometer fell to 11° below zero. Ice formed in the bedding, and soup froze upon the table. Every day new Coast passed before the eyes of the party, but around them the same interminable ice. By the 20th, they had reached the latitude of 75° 25′—a latitude never before attained in that meridian by keel of Christian ship.

We were borne along, said the lecturer, like specks upon a vast floating raft towards the unknown North! without possibility of escape or rescue, or even effort, and without the poor chance of leaving on the shore some hurried memorial to tell where we had gone. We spoke little of these things to each other; but the reflection could not be avoided. How likely it is that Sir John's vessels may have traveled as we are doing! How possible that our fates may be the same.

Dr. Kane thinks it probable that Franklin and his party were thus borne off to the North. He says:

It really seems as if the ice had suddenly opened to the North, and that Sir John, with his daring and energetic promptitude, had pushed into this new water, without delaying to give to the world behind him a notice of his course. Certain it is that the deserted encampment bears marks of hasty departure. If then, Franklin passed to the North, continued Dr. K., why has he not returned? The answer is conjecture. The treacherous leads may have closed upon him as they did upon us. He may have been borne as we were, imbedded in some vast ice-field. The same wind that forced the *Advance* and its surrounding ice-raft to the latitude of 75° 25′, may have blown upon him a few days longer than it did upon us. Or, more fortunately perhaps, at the outset, he may have found the water lead still open before him. In either case, a few weeks—it may be days—of progress, and he must have entered upon that dark and unknown water, which tinged our last winter's horizon as we floated on his track.

It is now six years since he passed beyond the recorded frontier of our world. What has been his fate: or rather *can he have* survived ? The consideration of this question was made exceedingly interesting by the lecturer. The casualties of Arctic navigation, though frequently disastrous, are not generally attended by the destruction of life. The ice-masses which crush by the lateral pressure of their incumbent weight, almost always give notice of their approach, and not unfrequently bridge the way for escape. Storms of wind are comparaiively rare ; and even when they do occur, the ice which destroys the whale ship, is almost the certain refuge of her crew. In the memorable gale of 1832, of the 1,000 seamen whose vessels were totally demolished, but seven lost their lives. Besides, vessels sailing in company, avoid as far as possible such a proximity as would expose both to the same peril at the same moment. The simultaneous destruction of the *Erebus* and *Terror* therefore, the Doctor looks upon as *not at all possible.*

Nor is there much reason to apprehend that the missing party has perished from cold, or starvation, or disease.—The *Igloe*, or snow-house, of the Esquimaux is an excellent and wholesome shelter. The servants of the Hudson's Bay Company preferred it to the winter hut, and for clothing, the furs of the Polar regions are better than any of the products of Manchester. The resources which that region evidently possesses for the support of human life are certainly surprisingly greater than the public are generally aware. Narwhal, white whales and seal—the latter in extreme abundance—crowd the waters of Wellington channel ; indeed, it was described as a region "*teeming with animal life.*" The migrations of the eider duck, the brant goose, and the auk—a bird about the size of our teal—were absolutely wonderful. The fatty envelope of these marine animals, known as *blubber*, supplies light and heat, their *furs*, warm and well-adapted clothing, their *flesh* wholesome and *anti-scorbutic* food.—The reindeer, the bear and the fox also abounded in great numbers, even in the highest latitude attained. In a word, Dr. K. announced that, after a careful comparison of all the natural resources of this region, he was convinced that food, fuel and clothing—the three great contributors to human existence—were here in superabundant plenty.

Dr. Kane does not suppose that the sea towards which his party was drifting, and from which, if they had reached it, no tidings of them would, in all probability, ever have returned, is literally an *open*, but comparatively an *iceless* sea. And he conjectures, that it is in this region, not far to the north and west of the point which the American Expedition reached, that Sir John Franklin and his companions are now probably immured; surrounded by seal, and the resources before described, but unable to leave their hunting ground and cross the "frigid Sahara," which intervenes between them and the world from which they were shut out.

In the following extract a thrilling and sublime incident is graphically described:

New Year's day, exactly one year ago, (continued Dr. K.) we found ourselves entering Baffin's Bay. Including our march up Wellington, we had drifted about four hundred miles. The premonitory cracks (fissures) had now opened into black rivers, traversing the ice for miles around like ramifying arteries. Everything pointed to our expected ice battle.

One of these great rivers, nearly as wide as the Schuylkill was astern of us, and over it a few nights' congelation had spread a film nearly a foot in thickness. That night I use the word artificially, for it was *all* night with us—of the 13th, after repeated "alarms," we were stretched out upon our buffalo robes with our knapsacks at hand, when the officer on deck called to us to hasten up. The thermometer was 40° below zero—70° below the freezing point; but the night, clear and starry, enabled us to penetrate the darkness to some distance astern. A white mass, seemingly in the air, was moving, with steady march, directly upon our brig. This we knew to be the crest of a gigantic *hummock*, its ridge of crumbling ice as white in the contrasting darkness, as the foam of rolling serf. Accompanying it was the solemn orchestra of ice voices, the booming diapason of the compressed floes. Presently came the mysterious cessation of these noises. The clamor ceased. We heard each other speak. A moment after came the well-known renewal,—the puppies, the shrieks and the locomotives. On came the crest; and now tumbling from it, we could see the heavy blocks of ice and hear their hollow coughs upon the snow-padded floes. Nearer yet we could define its masonry, and feel the transmitted undulation of the six foot ice, which, powerful as it was, formed no barrier to its advance. Now, to our quivering ship, came a vibratory trembling that made our lips tingle, as in a cotton factory at home. The colossal mass bears down upon us—closer—six yards—three yards—six feet—it ceases: its pulse had beaten, and the mysterious interval [of silence and quiet] had arrived. All that night we waited for its renewal; but the renewal never came. Five months afterwards that great ridge of ice stood in the same position beside us, a monument of God's mercy and man's own helplessness.

Dr. Kane gave a vivid account of the most remarkable of the Arctic phenomena, from which we quote the following as possessing special interest for the physician:

The cold came upon the voyagers gradually, and by habit they were enabled to keep as warm as necessary, without fires, for weeks after the thermometer was several degrees below zero. In the second week of September, the water casks froze up, and it became necessary to quarry out the ice and melt it before it could be used. By-and-bye, the waters of the sea congealed around them, and they were glued up in fixed ice. Moisture began to be a rarity, everything being frozen perfectly dry. The opening of a door was followed by a gust of smoke-like vapor, and outside every smoke-pipe exhaled purple steam. All their eatables froze into a mass of laughable solidification. Sugar was soon cut with a saw, butter with a chisel, and beef with an axe and crowbar!

The "crawl," the chill, the sensation of "cold" which at home is a temporary change of state, was here unknown—cold, of a highly wrought intensity, the one unvarying condition! When the mercury froze, and the alcoholic thermometers fell below—50°, and 80 odd below the freezing point, regular inspections took place during and after the walks of the men. A white spot on nose, lip or cheek, was the signal for a most uncharitable rubbing with snow; and many a time poor Jack, when pining for a warm stove, has been obliged to take, instead, a course of medical friction, with compulsory exercise. On one occasion a poor fellow, recovering from an attack of inflammation ot the lungs, was asked by his doctor how a certain frost-bitten ear came on? "Why," said he, producing a carefully folded scrap of an old newspaper, "I did'nt want to trouble you, Doctor; it dropped off last week; here it is." But the most depressing feature of their Arctic winter was the darkness of its long night, when for eighty days the sun was not visible. During this season the Aurora Borealis was an almost nightly visiter. The Aurora of the far North, however, is not the splendid display, either of illumination or color, or movement, which we see in the more southern latitudes; it resembles a white moonlit cloud, impressed clearly against the pure blue of the sky. Many other interesting phenomena of the Arctic night were described by the lectu rer

At length the sun returned, gradually and slowly, until on the 10th of April, the night was over, and the long Arctic day had commenced. But the return of the sun brought no additional warmth. On the contrary, the augmented evaporation and dryness were accompanied by a greater intensity of cold.

During the months immediately following the return of the sun, the entire horizon seemed lifted up and indefinitely extended. You saw on every side an inclined plane —vast and interminable except by the aerial limits of distance. Another form more startling, because more circumscribed, was that of a great circus. You looked as from the apex of a hollow cone, up a great encircling talus, whose summit was crowned by a steep and well-built wall. This effect was strangely impressive. The beholder was in the midst of a vast, apparently artificial, arena, whose centre, walk where you would, was still yourself; and whose walls, always there, conveyed the idea of a movable prison.

Among several instances of refraction related, was the spectral land off Cape Adair. On the evening of the 10th of February, while looking over the waste of snow, a flame-like streak, some 18° in length, was seen playing a short distance above the South-eastern horizon. Soon after, from its lower edge, depended a range of rude black knobs, which quickly assumed the shape and appearance of a range of hills, hanging inverted in the air; while, at the same time, a corresponding set, not inverted, rose to meet them from below—their bases remaining beneath the horizon. The appearance of these mountains meeting at their tops was such as to make the valleys between them assume the aspect of great tunnels. The land thus resurrected was more than 90 miles distant.

On the 5th of June, as if by some miraculous agency, the ice suddenly broke up, and in twenty minutes from the first alarm, the vessels were in a sea of tumultuous ice.—Five days afterwards they shook the free waters from their bows, and plunged along in a heavy sea-way, after an imprisonment of two hundred and sixty-seven days, and a drift of 1060 miles.

